# Supplementary material for: Beta cell reactivity defines disease-relevant pancreatic CD8 T cells in type 1 diabetes
Source: bioRxiv. 2026 Jun 10:2026.06.08.729940. Preprint. [Version 1] doi: 10.64898/2026.06.08.729940 (PMC13277807; doi:10.64898/2026.06.08.729940)
Supplement: Supplementary file 1 [file NIHPP2026.06.08.729940v1-supplement-2.pdf]

Supplementary Figure 1 (page 1)

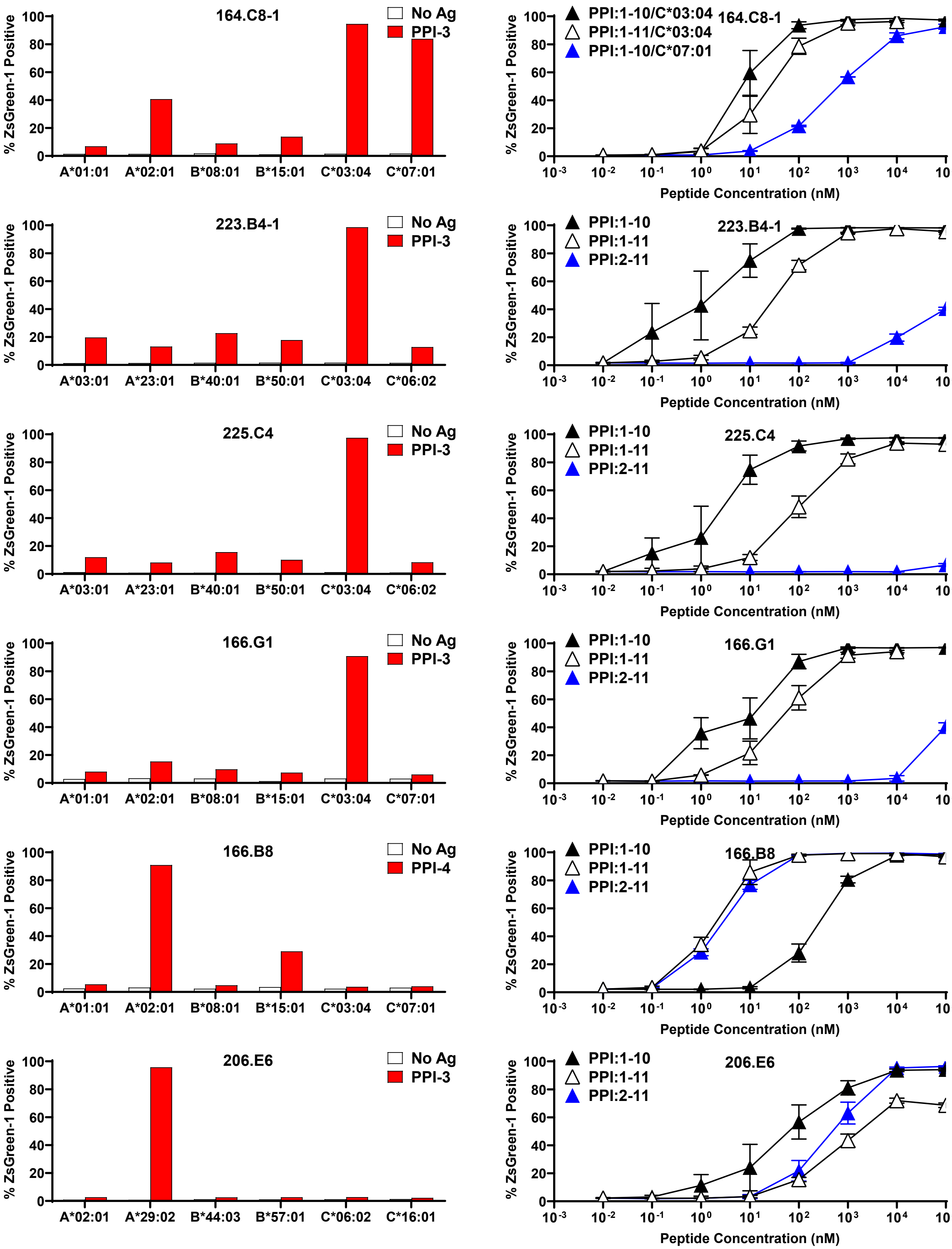

Supplementary Figure 1 (page 2)

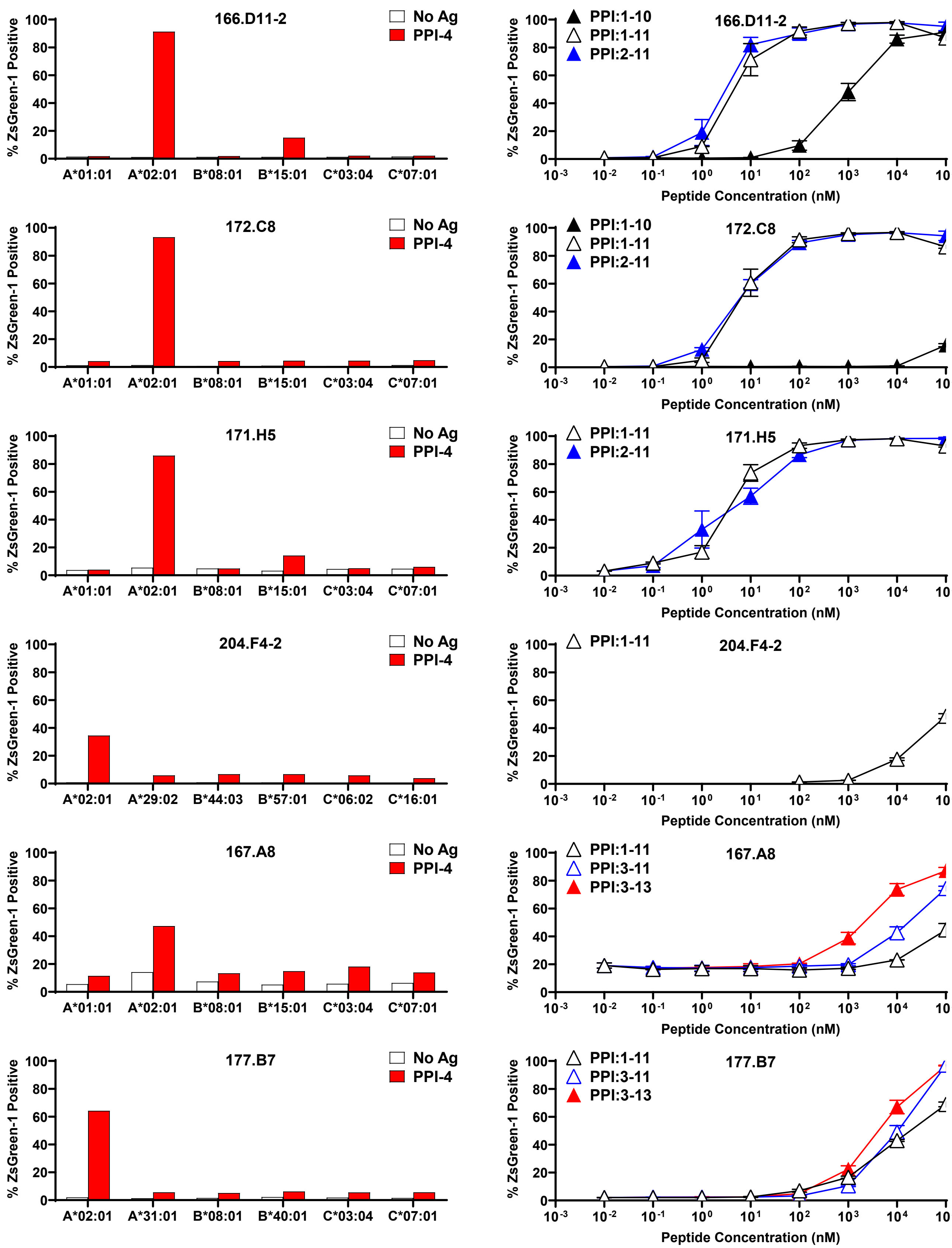

Supplementary Figure 1 (page 3)

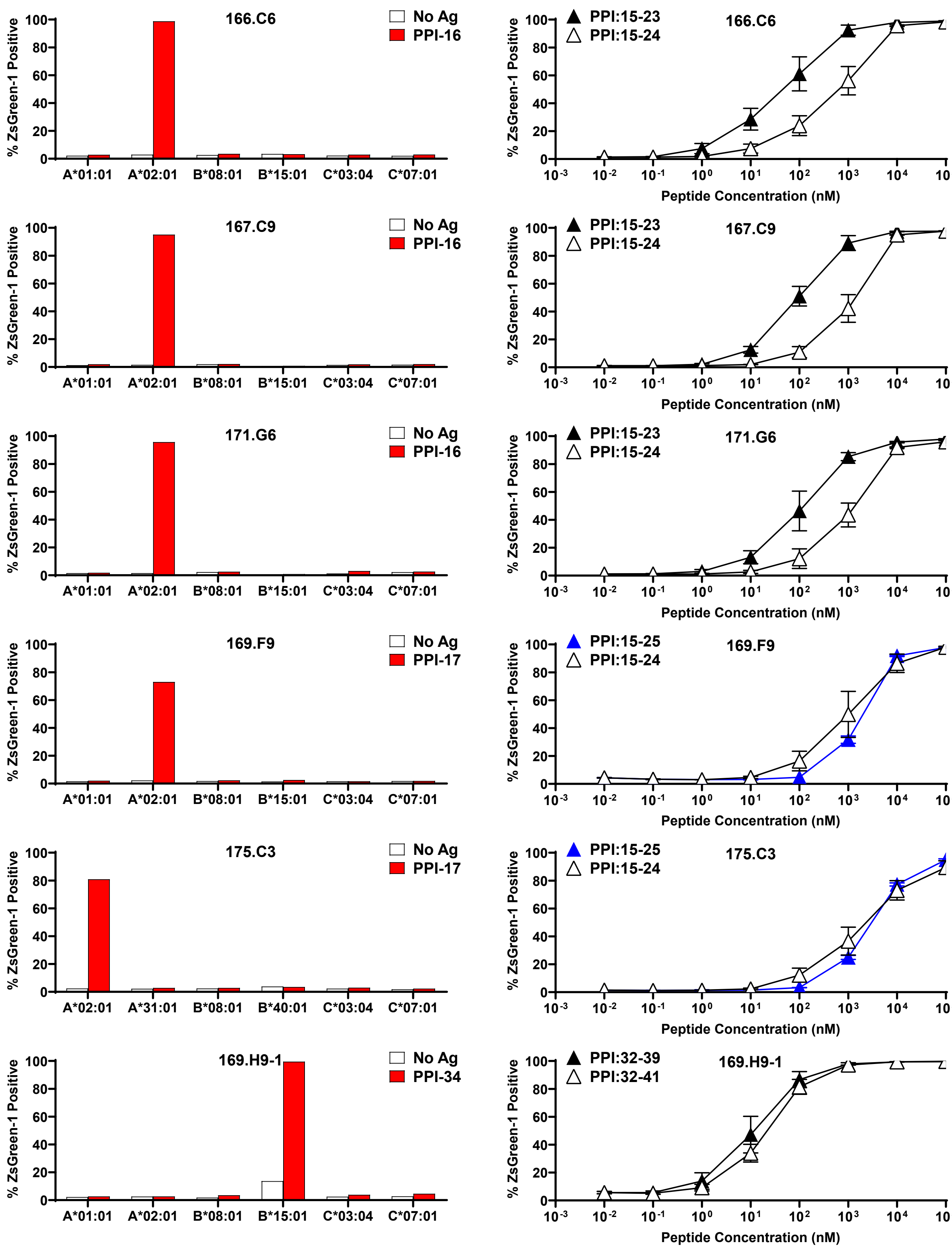

Supplementary Figure 1 (page 4)

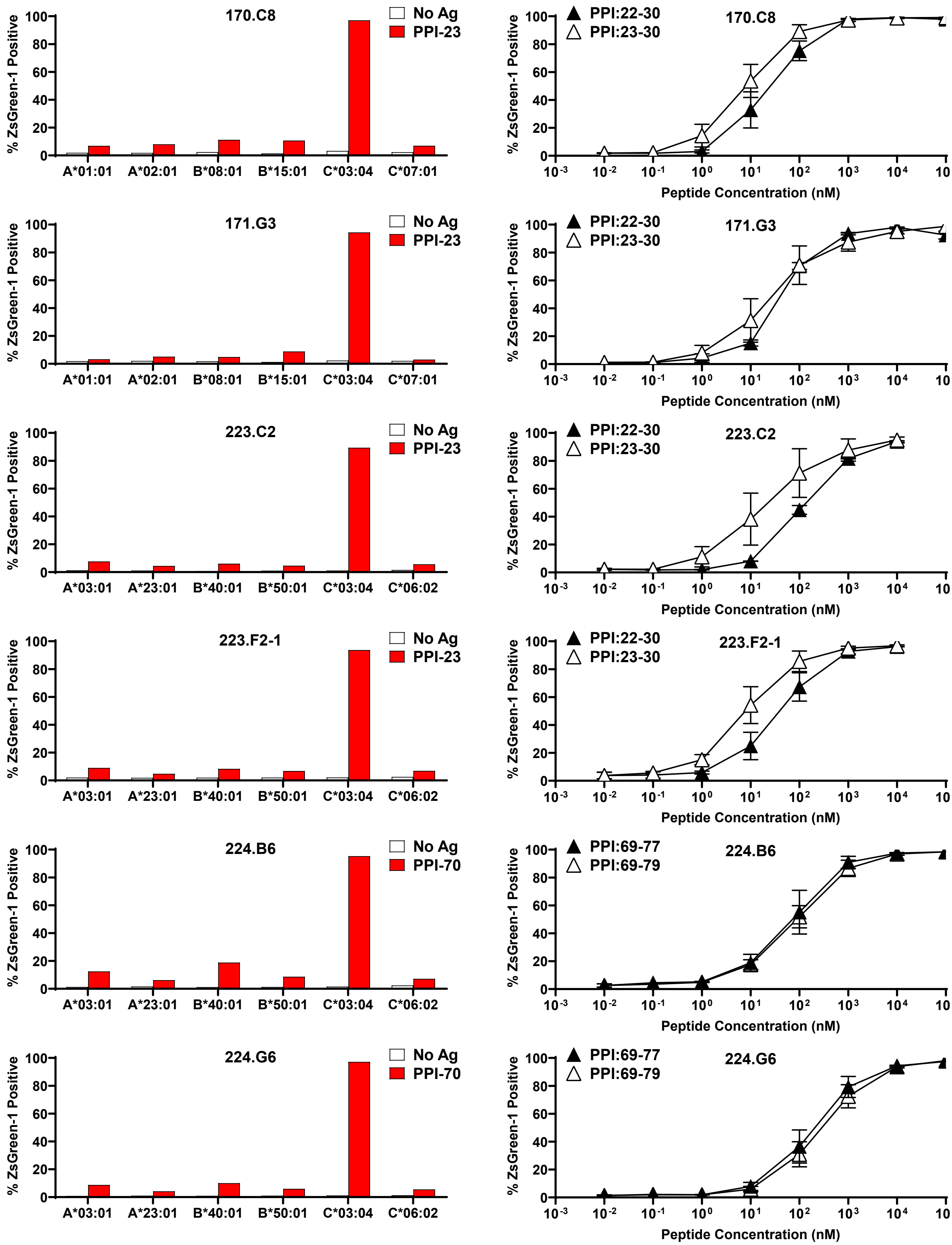

Supplementary Figure 1 (page 5)

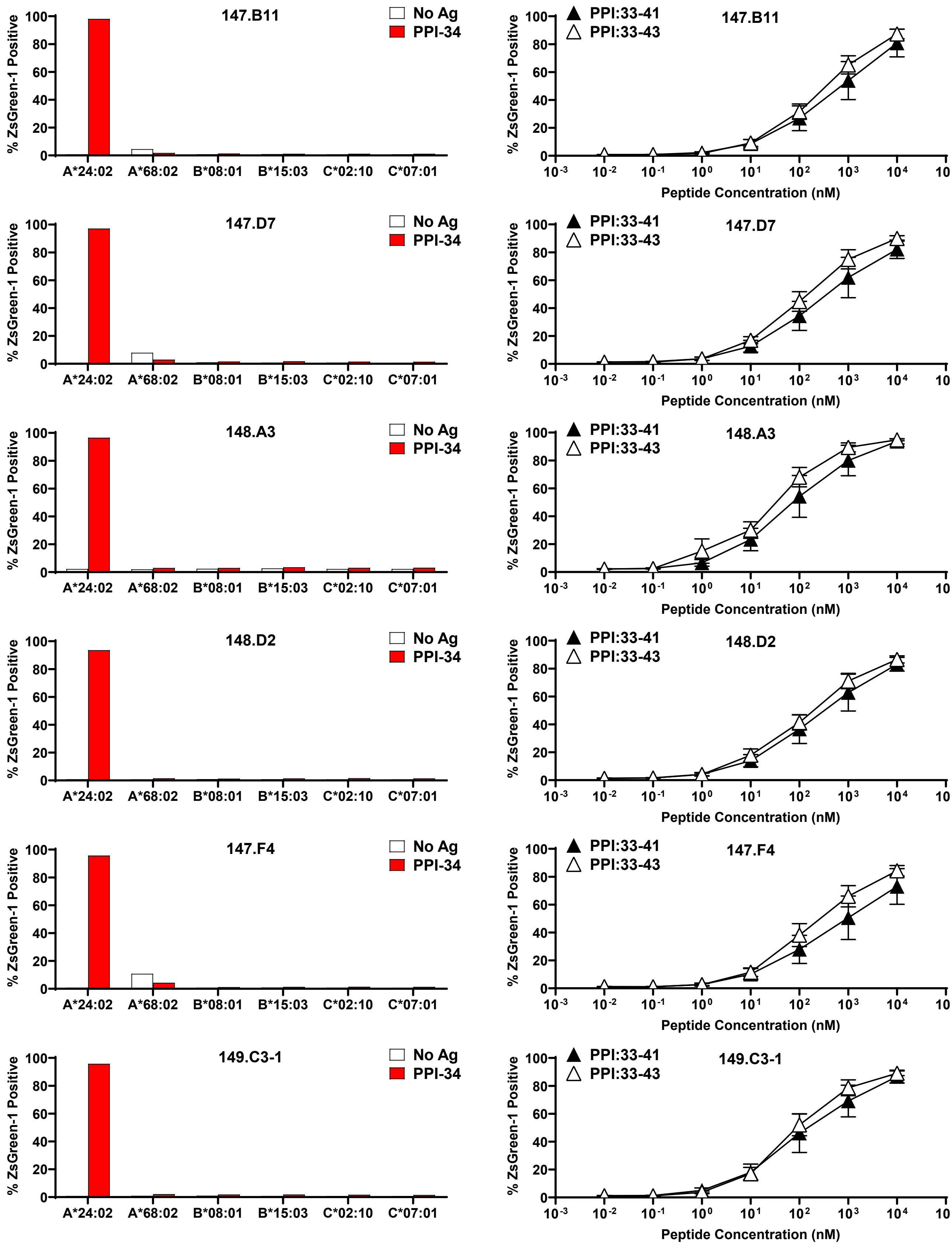

Supplementary Figure 1 (page 6)

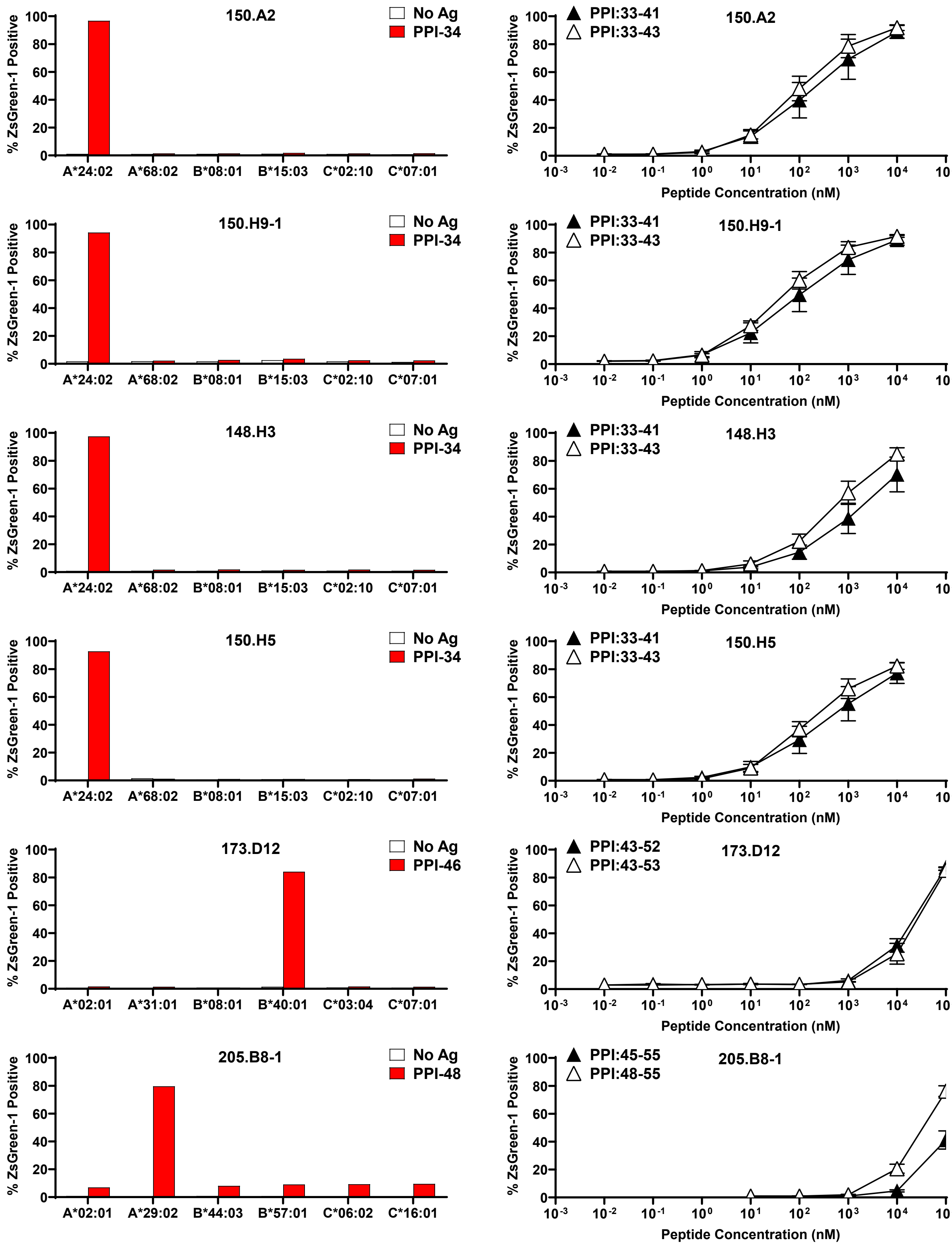

Supplementary Figure 1 (page 7)

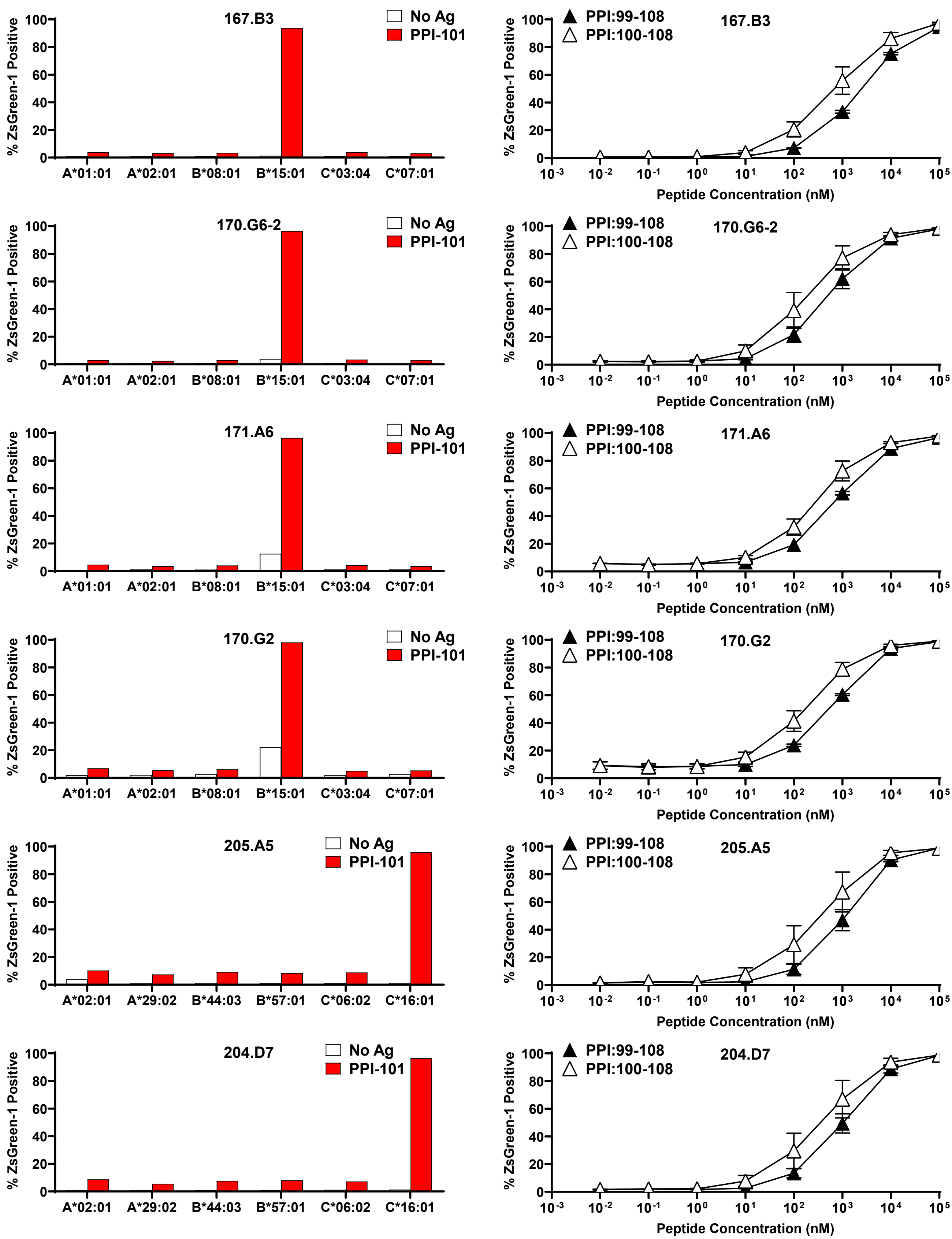

Supplementary Figure 1 (page 8)

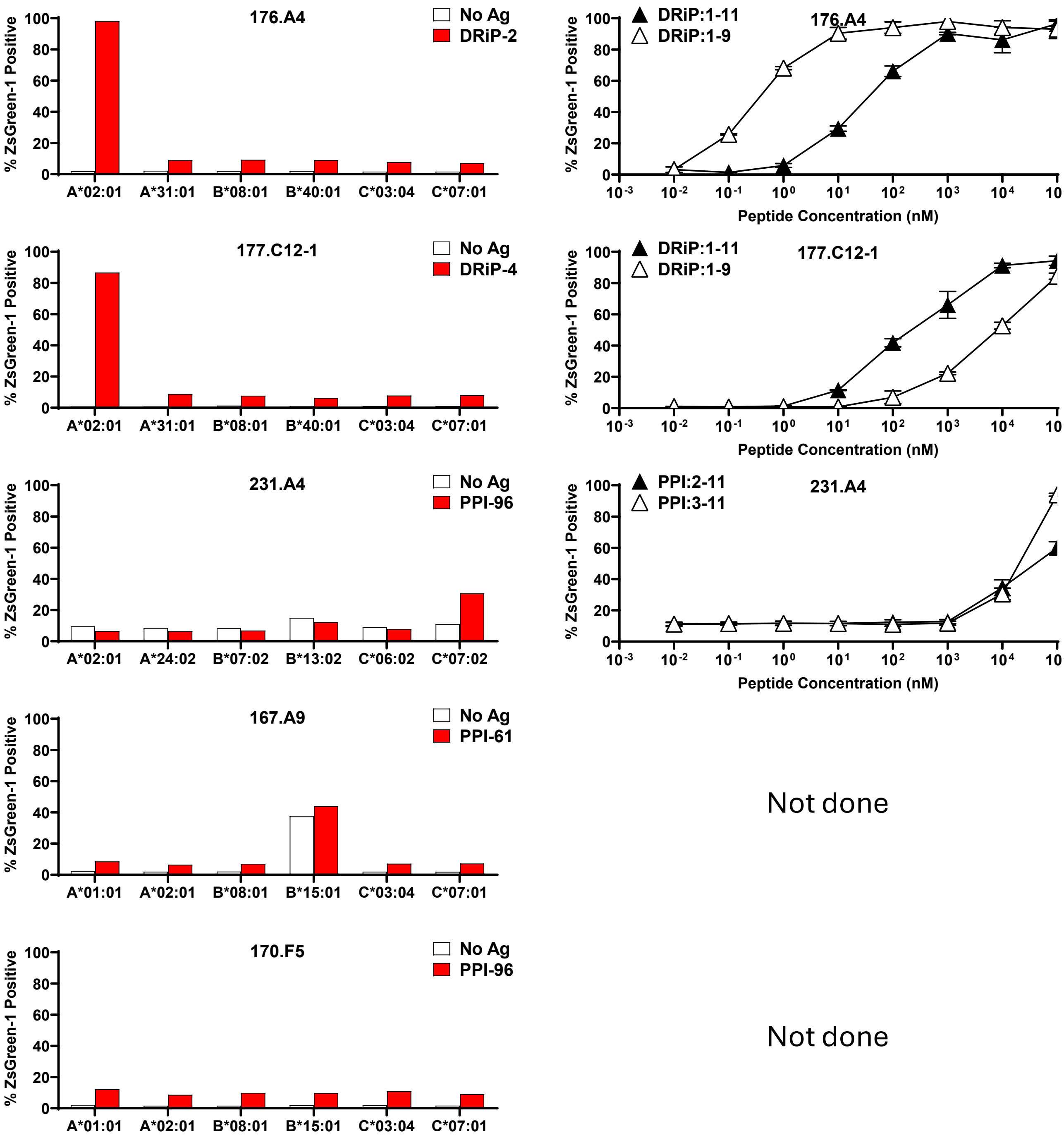

Supplementary Figure 1. HLA restriction and titration analysis of PPI- and insulin-DRiP-reactive TCR transductants. Left panels: TCR transductants that responded to truncated peptide pools in the initial screen were tested using K562 cells expressing single donor-matched HLA class I molecules to confirm peptide specificity and define HLA restriction. Responses are shown as the percentage of ZsGreen1-positive cells following peptide stimulation.

Right panels: For TCRs showing peptide-HLA-specific responses, TCR transductants were stimulated with cognate peptides from the corresponding pools across a range of concentrations in the presence of K562 cells expressing the defined HLA class I molecules. Activation was assessed by ZsGreen1 expression driven by an NFAT reporter, and mean values ± SEM from two or three independent experiments are shown.

Supplementary Figure 2 (page 1)

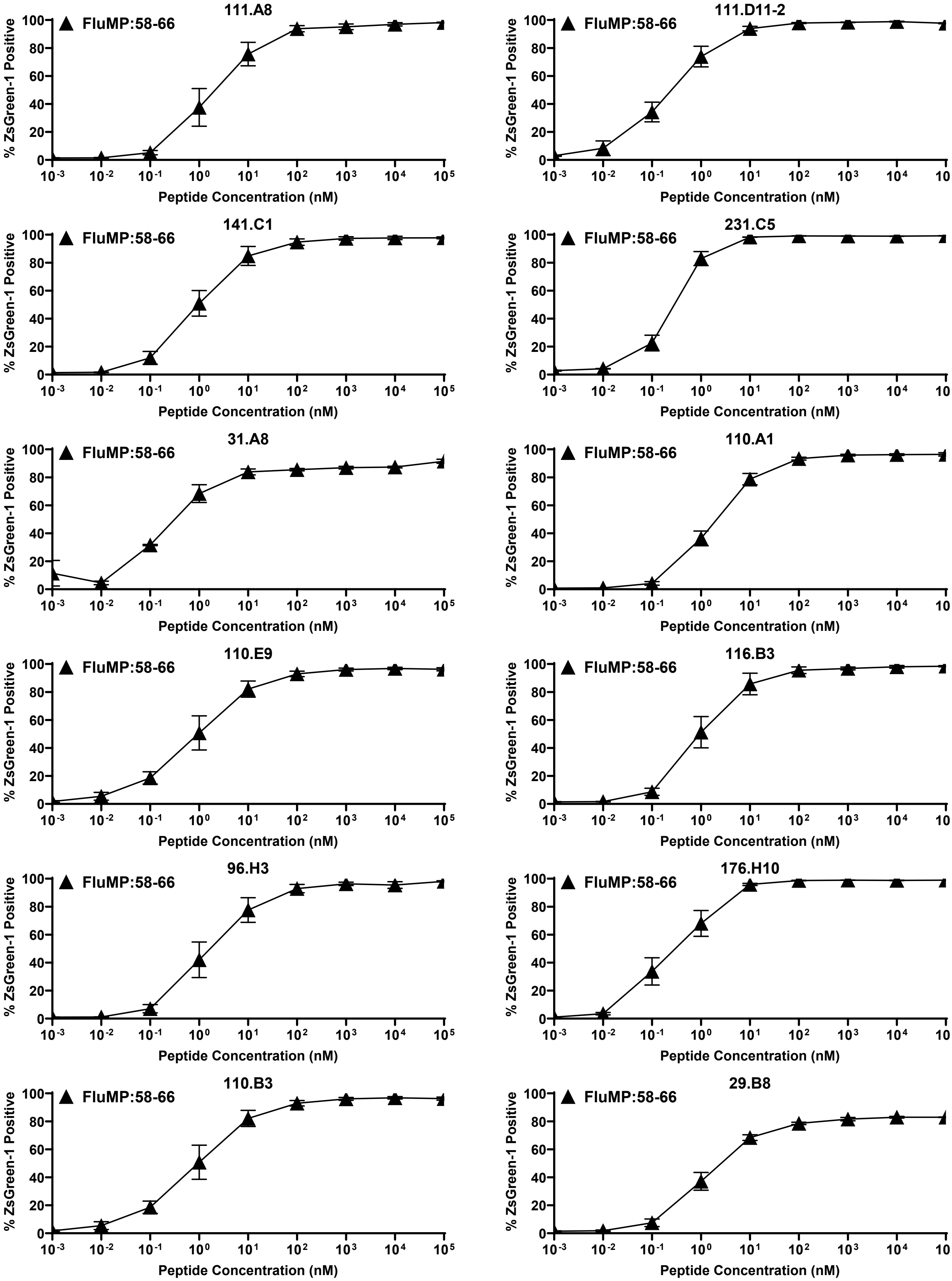

Supplementary Figure 2 (page 2)

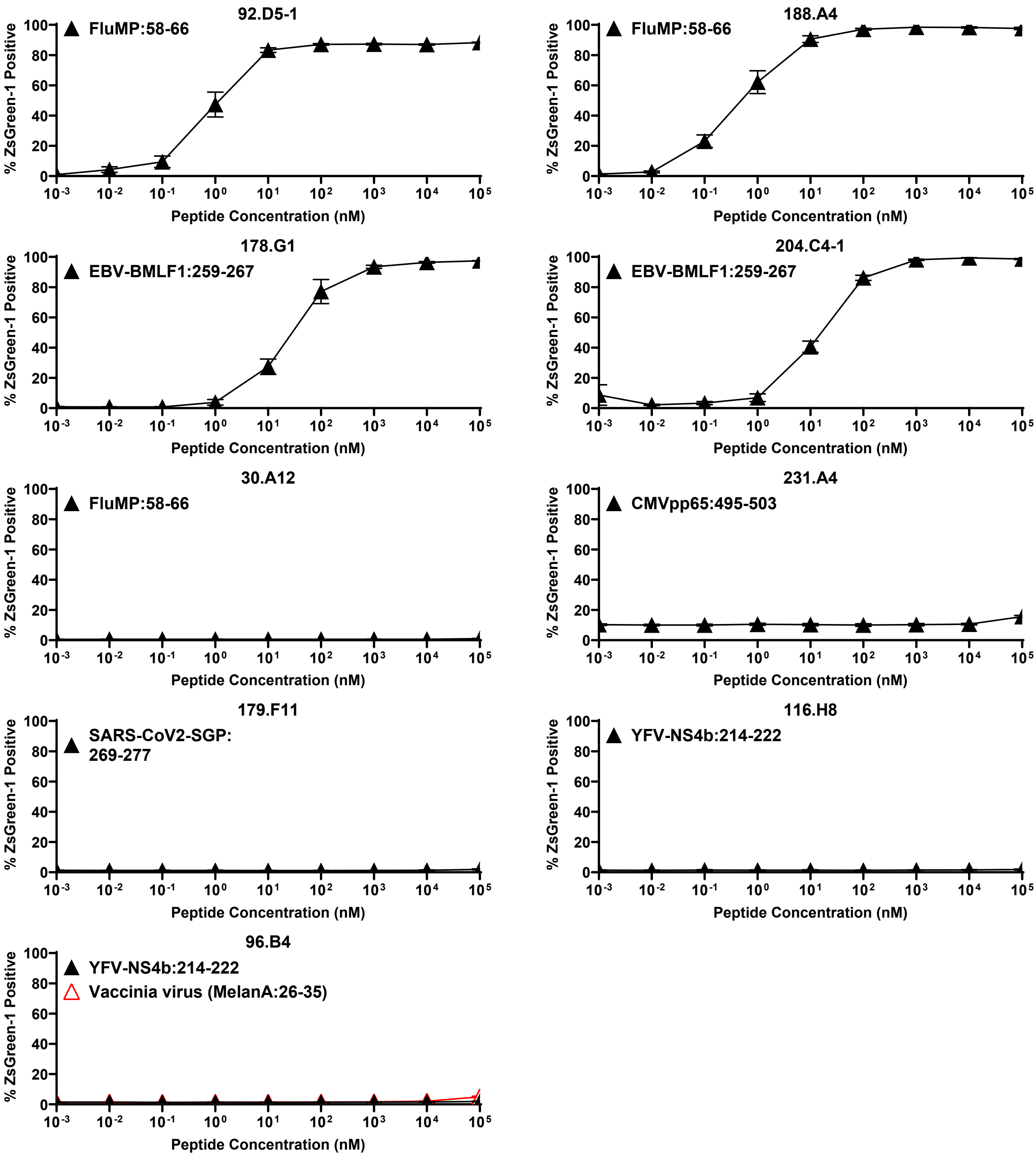

Supplementary Figure 2. Titration analysis of transductants expressing public TCR clonotypes. Transductants expressing public TCR clonotypes identified from the pancreas were stimulated with cognate peptides reported in the Immune Epitope Database (IEDB) across a range of concentrations in the presence of K562 cells expressing the corresponding HLA class I molecules. Activation was assessed by ZsGreen1 expression driven by an NFAT reporter, and mean values ± SEM from three independent experiments are shown.

Supplementary Figure 3

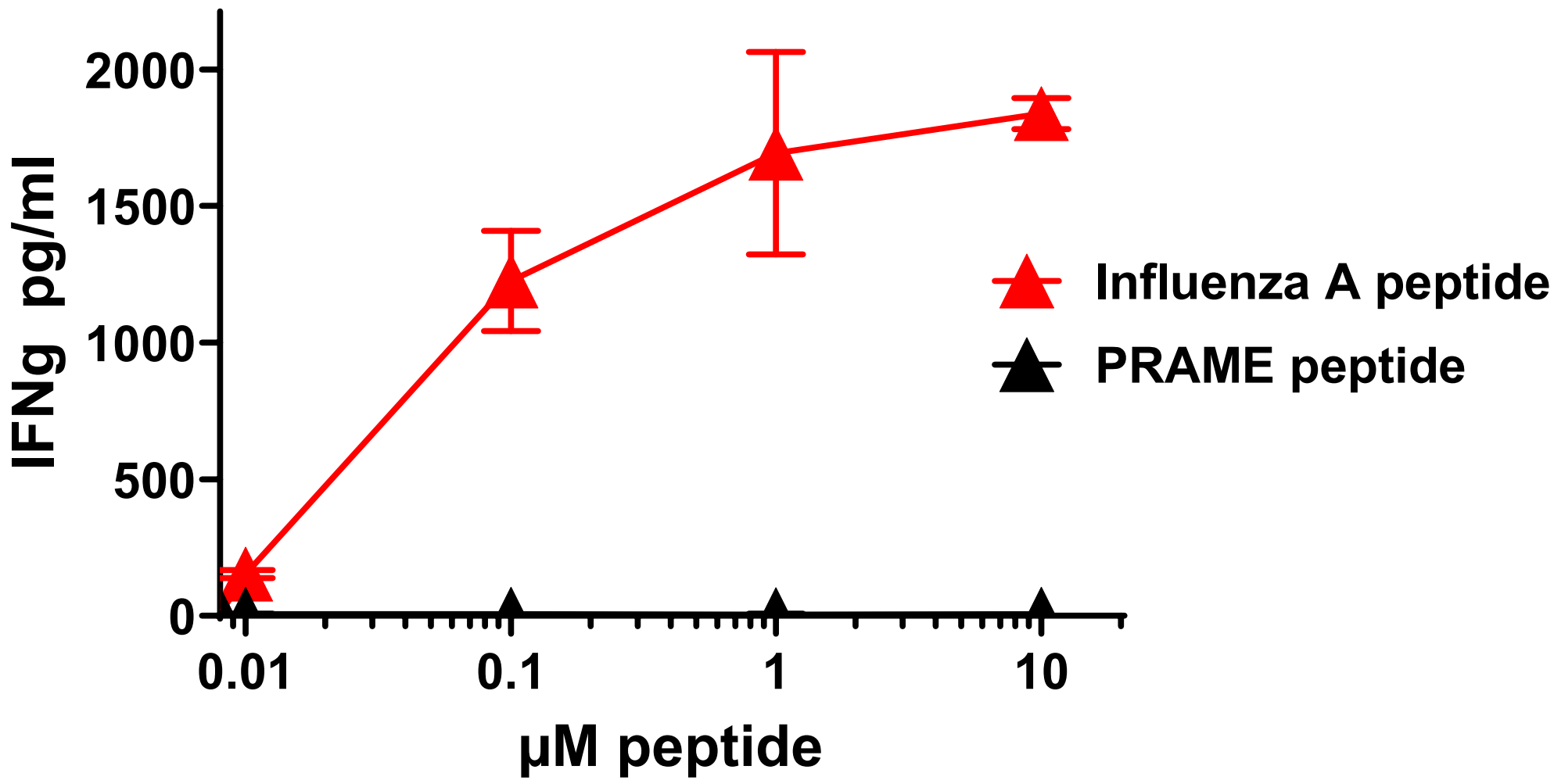

Supplementary Figure 3. Influenza A peptide reactivity of islet-derived T cell clones from a T1D donor. Two T cell clones were established from T cell lines by 0.3 cells/well limiting dilution cloning derived from two individual islets hand-picked from enzyme-dispersed pancreas slice samples from an nPOD donor 6472 (10.25-year-old female with 4 years duration of T1D; RRIS:SAMN15879525). T cell lines that responded to the influenza A peptide were cloned by limiting dilution. Clones were cultured with the influenza A peptide (GILGFVFTL) or an islet-irrelevant control peptide, Preferentially Expressed Antigen of Melanoma ($PRAME_{425-433}$ SLLQHLIGL), in the presence of HLA-A2-expressing C1R cells. Interferon-gamma secretion in culture supernatants was measured by ELISA. Experiments were performed in triplicate; representative results from one of the two clones are shown.

Supplementary Figure 4 (page 1)

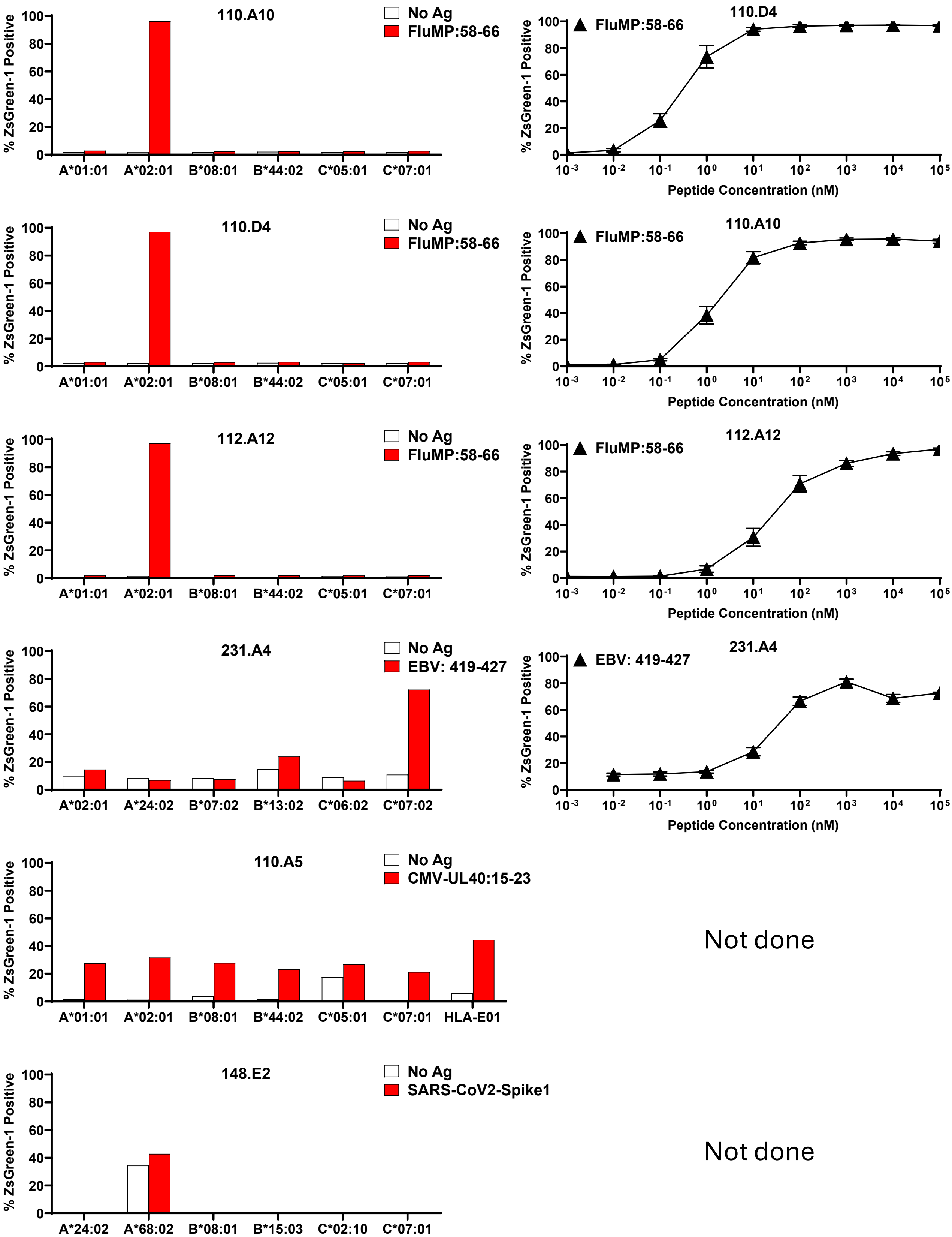

Supplementary Figure 4 (page 2)

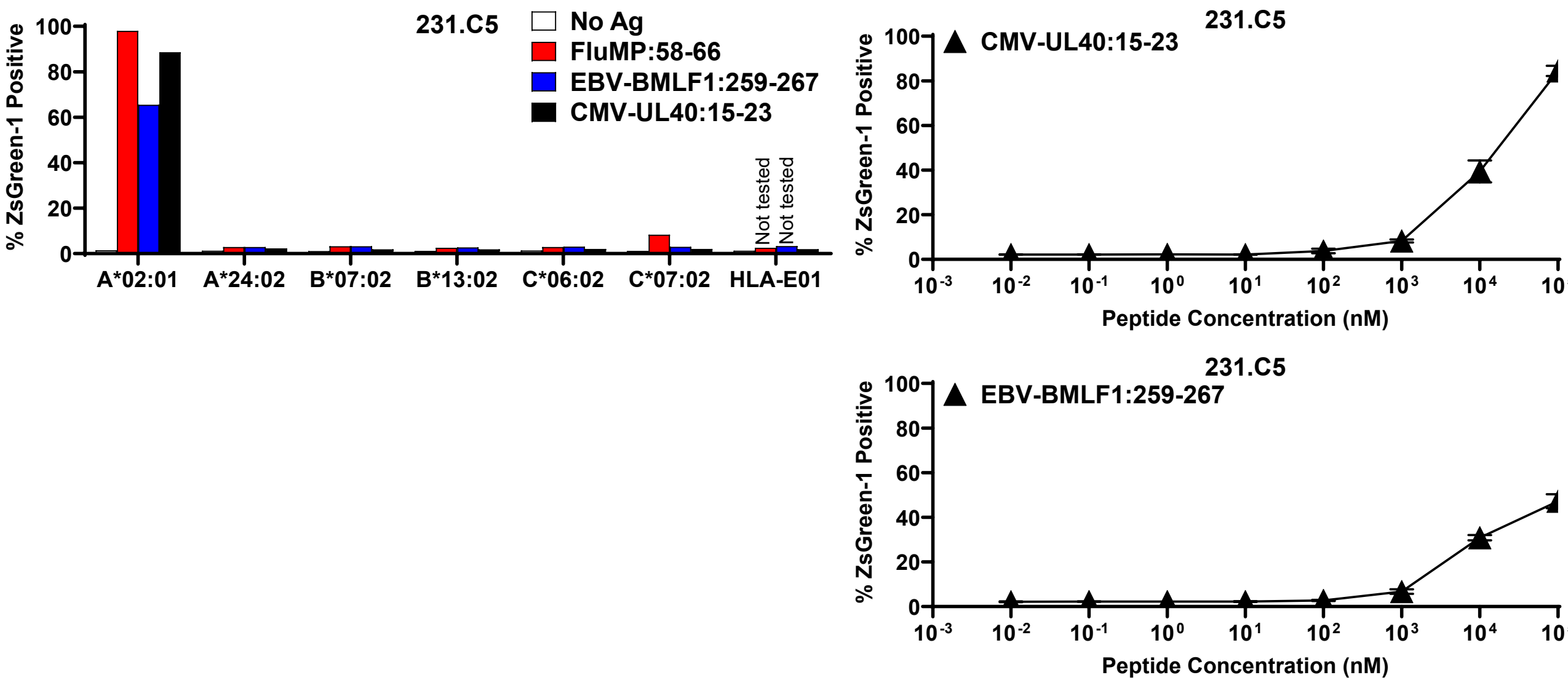

Supplementary Figure 4. HLA restriction and titration analysis of viral peptide-reactive TCR transductants. Left panels: TCR transductants that responded to viral peptides in the initial screen were tested using K562 cells expressing single donor-matched HLA class I molecules to confirm peptide specificity and define HLA restriction. Responses are shown as the percentage of ZsGreen1-positive cells following peptide stimulation.

Right panels: For TCRs showing peptide-HLA-specific responses, TCR transductants were stimulated with cognate peptides across a range of concentrations in the presence of K562 cells expressing the defined HLA class I molecules. Activation was assessed by ZsGreen1 expression driven by an NFAT reporter, and mean values ± SEM from three independent experiments are shown.

Supplementary Figure 5

T1D donors

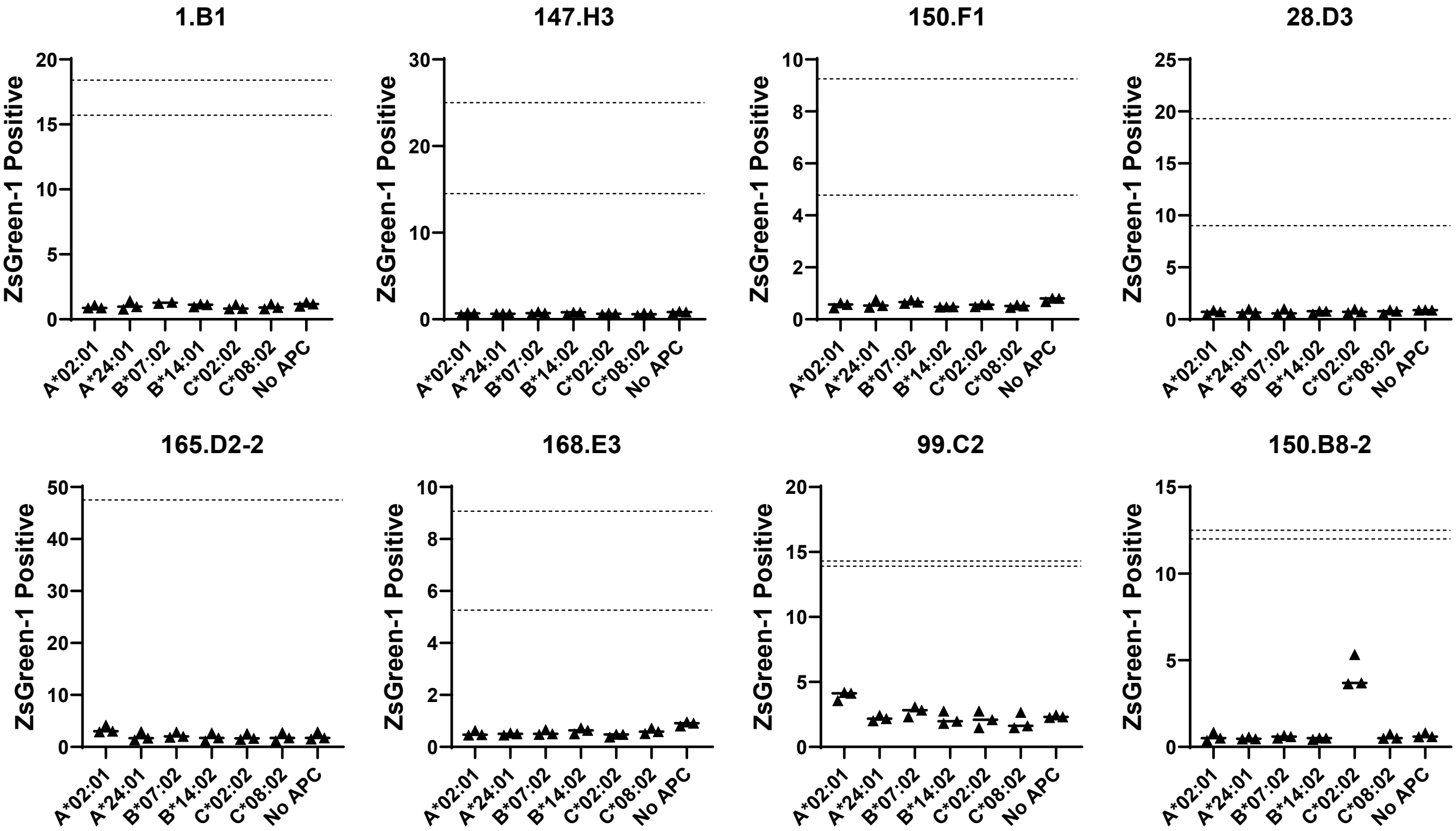

GAD antibody-positive and non-T1D donors

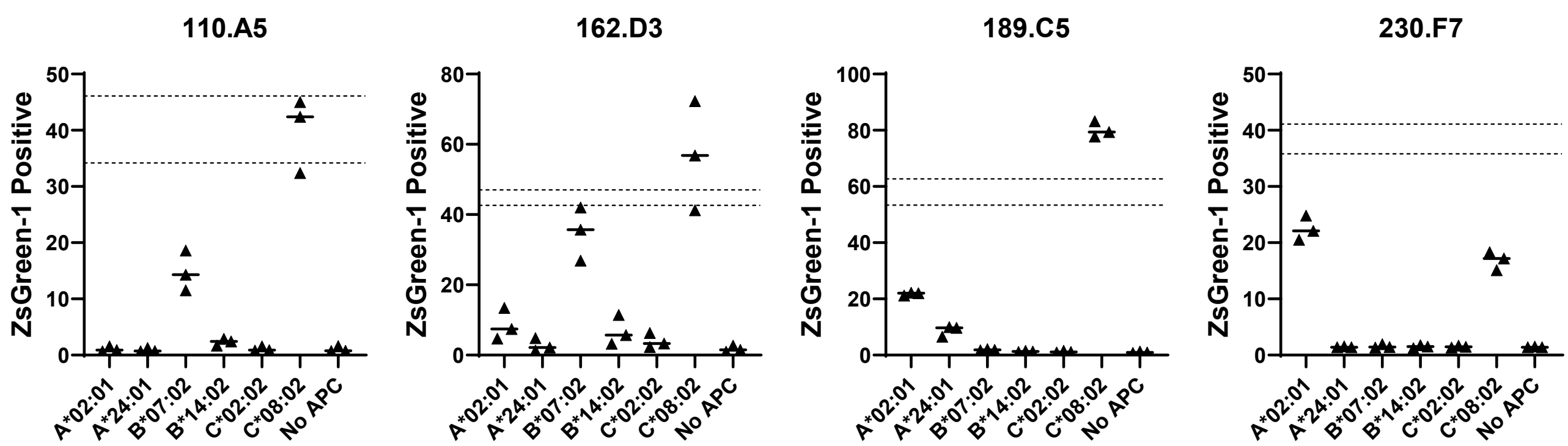

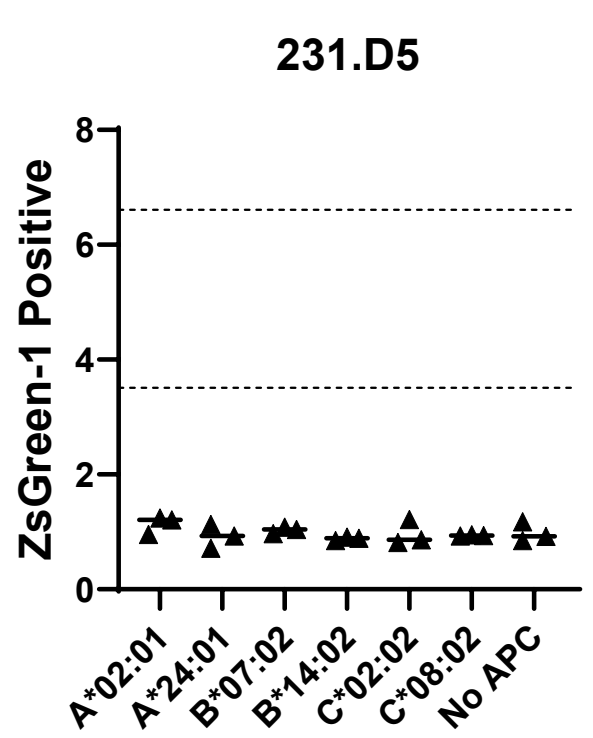

Supplementary Figure 5. Testing allogeneic responses of sBC-reactive TCR transductants. TCR transductants defined as reactive to sBCs were tested for responses to K562 cells expressing individual HLA class I molecules carried by the sBC donor to assess potential alloreactivity. Activation was assessed by ZsGreen1 expression driven by an NFAT reporter, and mean values ± SEM from three independent experiments are shown.
